# Cavity-Tuned Exciton Dynamics in Transition Metal Dichalcogenides Monolayers

**DOI:** 10.3390/ma17164127

**Published:** 2024-08-20

**Authors:** Kaijun Shen, Kewei Sun, Maxim F. Gelin, Yang Zhao

**Affiliations:** 1 School of Materials Science and Engineering, Nanyang Technological University, Singapore 639798, Singapore; 2 School of Science, Hangzhou Dianzi University, Hangzhou 310018, China

**Keywords:** variational method, coherent states, Davydov ansatz, transition metal dichalcogenides, nanocavity, exciton dynamics, time-resolved fluorescence

## Abstract

A fully quantum, numerically accurate methodology is presented for the simulation of the exciton dynamics and time-resolved fluorescence of cavity-tuned two-dimensional (2D) materials at finite temperatures. This approach was specifically applied to a monolayer WSe_2_ system. Our methodology enabled us to identify the dynamical and spectroscopic signatures of polaronic and polaritonic effects and to elucidate their characteristic timescales across a range of exciton–cavity couplings. The approach employed can be extended to simulation of various cavity-tuned 2D materials, specifically for exploring finite temperature nonlinear spectroscopic signals.

## 1. Introduction

Monolayer transition metal dichalcogenides (TMDs) are celebrated for their broken inversion symmetry, resulting in a non-zero Berry curvature and robust valley–spin interactions. Such valley–spin interactions enable selective valley polarization through optical or electromagnetic means [1,2]. The reduction in the layer number results in the transformation of TMDs from indirect to direct band gap semiconductors [3,4]. This layer-number-dependent symmetry variation also allows for the targeted manipulation of valley properties, establishing TMDs as a pivotal platform for valleytronic device development. In tungsten-based monolayers, low-temperature photoluminescence studies have shown that dark exciton states [5,6,7,8], which are momentum-forbidden and significantly influence the optical and electronic behavior of TMDs [9,10,11,12], exhibit lower energy levels than their bright counterparts.

Additionally, the interaction of two-dimensional (2D) materials with light, crucial for devices such as nanolasers [13,14], can be enhanced by coupling these materials with optical cavities or through intrinsic polaritonic resonances [15,16]. The strong interaction between light and matter within optical cavities leads to the creation of quasiparticles termed polaritons. In organic microcavities, when the vibronic coupling between excitons and phonons is comparable in strength to the exciton–photon coupling, multiple polariton branches have been experimentally observed [17,18] alongside phonon-assisted relaxation processes [19,20]. Recently, these phenomena have been directly utilized to investigate polaritonic nonlinearities at the single-photon level [21]. These entities underpin a wide array of both quantum and classical behaviors across various materials and spectral regions, such as polariton lasing [22], polariton-induced blockade [23,24], and the Bose–Einstein condensation of polaritons [25]. Recent developments in 2D TMDs interfaced with photonic architectures have paved the way for detailed studies of exciton–polaritons on an atomic level. The valley-selected property of TMDs excitons, when utilized in hybrid photonic systems, has enabled the creation of valley-polarized exciton–polaritons [26]. Moreover, excitons located in the K and K’ valleys are interconnected through time reversal symmetry. Consequently, removing valley degeneracy in these systems might facilitate the study of topological exciton–polaritons that exhibit broken time reversal symmetry at visible frequencies [27]. Another compelling dimension of exciton behavior in 2D TMDs emerges when two monolayers are angularly misaligned and superposed, creating bilayer heterostructures [28,29]. The resulting moiré patterns lead to moiré excitons within individual layers, which show a pronounced increase in nonlinearity due to exciton blockade effects. This significant interaction between moiré excitons and photonic modes in cavities can produce polaritons with marked nonlinearity [30], forming an excellent platform for studying highly interactive exciton–polaritons. Such integration facilitates the development of compact, on-chip devices [31] by coupling excitons in TMDs with photonic modes within planar nanocavities [32,33], paving the way for novel light–matter interaction platforms.

Photoluminescence (PL) spectroscopy remains a fundamental technique for probing quantum phenomena and complex interactions in TMD monolayers [34]. At a low temperature, PL reveals the presence of bound excitonic complexes, such as biexcitons and trions [5,35,36,37]. The significant role of phonon-assisted recombination in shaping the PL emission characteristics can be highlighted, particularly within cavity-controlled materials, leading to asymmetrical line shapes and enhanced sidebands—a phenomenon termed cavity-coupled PL emission [31]. Time-resolved fluorescence (TRF) spectroscopy [38,39,40] further allows the examination of these interactions over brief intervals, providing deeper insights into exciton dynamics and relaxation processes.

Despite the utility of conventional theoretical approaches such as quantum master equations [31] and cluster expansion methods [5], these often fall short in scenarios involving strong exciton–photon or exciton–phonon interactions. However, the multiple Davydov Ansatz (mDA) technique has recently emerged as a robust, numerically precise method [41,42], applied to a diverse array of multidimensional challenges, including analyzing Landau–Zener transitions [43,44], ultrafast dynamics at conical intersections (CIs) [45,46], and hole–magnon dynamics in an antiferromagnet [47,48]. The mDA method is a variational method to solve the many-body time-dependent Schrödinger equation, where the wave function for bosonic degrees of freedom is expanded into multiple coherent states for each boson mode in a multispecies boson bath of arbitrary spectral density functions. If the multiplicity is high enough, the so-obtained solutions converge to an exact solution. Here, we employ the mDA framework, complemented by thermofield dynamics (TFDs) representation [49,50], to offer a comprehensive, microscopically accurate simulation method for excitonic dynamics and spectroscopic responses of cavity-tuned single-layer WSe2 at finite temperature and multiple exciton–cavity (EC) coupling strengths.

In Section 2, we establish a microscopic model of the WSe_2_ monolayer embedded in a microcavity and introduce the methodologies adopted. Section 3 is devoted to a comprehensive discussion of calculated exciton populations and TRF spectra for a range of EC coupling strengths from weak to strong. Conclusions are offered in Section 4.

## 2. Microscopic Model and Methodologies

**Figure 1:**
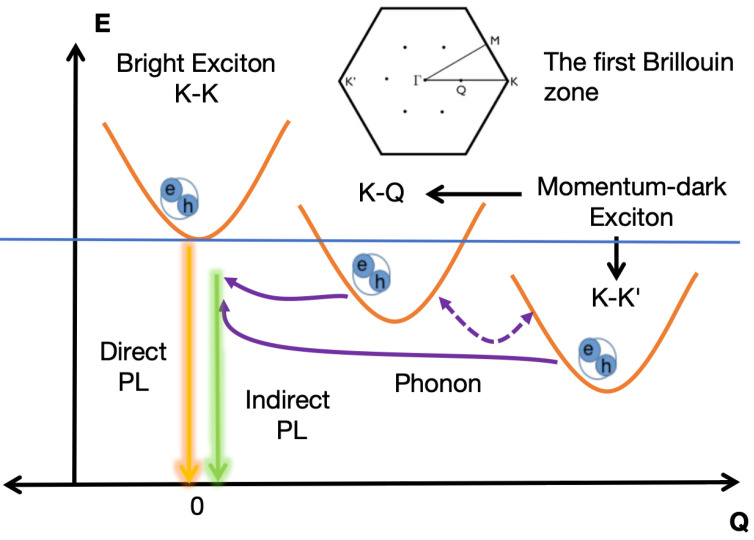
A graphical representation of the direct and phonon-assisted PL emission processes. The momentum–dark excitons located at the K−Q and $K-K^{\prime }$ points undergo radiative decay via absorbing or emitting a phonon, thus contributing to the indirect PL emissions. The inset in the upper middle part illustrates the distribution of conduction electrons across various valleys within the first Brillouin zone.

PL and TRF profiles of WSe_2_ monolayers are predominantly determined by KK, KQ, and ${\mathrm{KK}}^{\prime }$ excitons, of which the first K represents hole momentum in the valence band, and the second symbol indicates the electron momentum in the conduction band, corresponding to momenta at 1Γ, 2Λ, and 3K respectively. Excitons of higher energy than the above states have a negligible influence on the dynamic properties and optical signals at room temperature and below. The phonon-mediated PL is attributed to the KQ and ${\mathrm{KK}}^{\prime }$ intervalley dark states, which is in contrast to the KK intravalley excitons that are optically bright and yield direct PL emission. The contrasting dynamics of direct versus indirect PL are detailed in Figure 1. Following this qualitative picture, the exciton–polariton dynamics of the cavity-modified WSe_2_ monolayer can be described by a Hamiltonian containing three excitonic states, a cavity mode, and several dominant phonon modes [5,31,51,52]:

(1) $$\begin{array}{ccc} H & = & \sum_{\left(iQ_{\|}\right)}E_{\left(iQ_{\|}\right)}X_{\left(iQ_{\|}\right)}^{†}X_{\left(iQ_{\|}\right)}+\hbar \omega_{\sigma_{+}}c_{\sigma_{+}}^{†}c_{\sigma_{+}}\\ & & +M_{\sigma_{+}}\left[c_{\sigma_{+}}^{†}X_{\left(1\Gamma \right)}+c_{\sigma_{+}}X_{\left(1\Gamma \right)}^{†}\right]+\sum_{\alpha q_{\|}}\hbar \Omega_{\alpha ,q_{\|}}b_{\alpha ,q_{\|}}^{†}b_{\alpha ,q_{\|}}\\ & & +\sum_{ij\alpha Q_{\|}q_{\|}}D_{\alpha q_{\|}}^{ij}X_{\left(jQ_{\|}+q_{\|}\right)}^{†}X_{\left(iQ_{\|}\right)}\left(b_{\alpha ,-q_{\|}}^{†}+b_{\alpha ,q_{\|}}\right). \end{array}$$

Here, operators $X_{\left(iQ_{\|}\right)}^{\left(†\right)}$, $c_{\sigma_{+}}^{\left(†\right)}$, $b_{\alpha ,q_{\|}}^{\left(†\right)}$ ($X_{\left(iQ_{\|}\right)}$, $c_{\sigma_{+}}$, $b_{\alpha ,q_{\|}}$) create (annihilate), respectively, exciton states $\left(iQ_{\|}\right)$, circularly polarized photon states $\sigma_{+}$ and phonon modes α with momentum $q_{\|}$, and frequency $\Omega_{\alpha ,q_{\|}}$. $E_{\left(iQ_{\|}\right)}$ is the excitonic dispersion relation, where the symbol ‖ denotes the momentum’s in-plane component. The strength of coupling between the exciton and cavity photon modes is quantified by the coefficient $M_{\sigma_{+}}$. The tensor $D_{\alpha q_{\|}}^{ij}$, which maps the coupling of excitons with phonons, can be computed through the density functional theory (DFT) formalism (mean effective deformation potential approximation) [7,53]:

(2) $$\begin{array}{c} D_{\alpha q_{\|}}^{ij}=\sum_{k}\Phi_{k}^{i*}\left(\Phi_{k+q_{1\|}}^{j}g_{\alpha q_{\|}}^{ck(ij)}-\Phi_{k-q_{2\|}}^{j}g_{\alpha q_{\|}}^{vk(ij)}\right). \end{array}$$

where $\Phi_{k}^{i}$ is the excitonic wave function in the momentum space, which can be obtained by solving the Wannier equation. $g_{\alpha q_{\|}}^{c/vkij}$ are the electron–phonon interaction coefficients within the conduction and valence bands [54,55], where α and $q_{\|}$ define the phonon modes scattered between the excitonic band index *i* and *j*.

(3) $$\begin{array}{ccc} g_{\alpha q_{\|}}^{c/vk(ij)} & = & \sqrt{\frac{\hbar }{2M\Omega_{\alpha ,q_{\|}}}}\langle j,{k+q}_{\|}|\Delta V_{q_{\|}}^{\alpha }|i,k\rangle \\ & = & \sqrt{\frac{\hbar }{2M\Omega_{\alpha ,q_{\|}}}}\int_{\mathrm{UC}}\psi_{j,k+q_{\|}}^{*}\left(r\right)\Delta V_{q_{\|}}^{\alpha }\left(r\right)\psi_{i,k}\left(r\right)\;d^{3}r \end{array}$$

are the electron–phonon matrix elements in conduction/valence band involving integrals over the unit cell (UC). M encapsulates the total mass of atoms within a unit cell, while |i,k〉 describes the Bloch eigenstates $\psi_{i,k}\left(r\right)$ characterized by wave vector k and an excitonic band index *i*. The potential perturbation, $\Delta V_{q_{\|}}^{\alpha }$, is derived using density functional perturbation theory. The first-order deformation potential, or the acoustic deformation potential, is expressed as $\langle j,{k+q}_{\|}|\Delta V_{q_{\|}}^{\alpha }\left|i,k\rangle /\right|q_{\|}|$. It can be shown that acoustic phonons at the Γ do not contribute to the electron–phonon coupling. The zero-order deformation potential, also known as the optical deformation potential, is defined as $\langle j,{k+q}_{\|}|\Delta V_{q_{\|}}^{\alpha }|i,k\rangle$ [54]. Detailed parameters for this potential are documented in Ref. [56]. In this work, we neglect cavity energy losses, which can be realized in fabricated photonic crystal nanobeam (PhCnB) cavities with a notably high-quality factor [57]. For instance, the exciton–photon coupling of a thin excitonic material positioned at the center of a high-quality, symmetric Fabry–Pérot cavity near the cavity resonance can be fine-tuned by adjusting the mirror reflectivity and the cavity length [58,59,60].

To treat finite temperature effects with the TFD method, we introduce the extended Hamiltonian [61,62,63]:

(4) $$\begin{array}{c} \bar{H}=H-{\tilde{H}}_{\mathrm{ph}}=H-\sum_{\alpha q_{\|}}\Omega_{\alpha ,q_{\|}}{\tilde{b}}_{\alpha ,q_{\|}}^{†}{\tilde{b}}_{\alpha ,q_{\|}} \end{array}$$

where

(5) $$\begin{array}{c} {\tilde{H}}_{\mathrm{ph}}=\sum_{\alpha q_{\|}}\Omega_{\alpha ,q_{\|}}{\tilde{b}}_{\alpha ,q_{\|}}^{†}{\tilde{b}}_{\alpha ,q_{\|}} \end{array}$$

is the phonon Hamiltonian acting in the fictitious “tilde” vibrational space. Having performed the thermal Bogoliubov transformation, we arrive at the TFD Hamiltonian [49,50]:

(6) $$\begin{array}{cc} {\bar{H}}_{\theta } & =e^{iG}\bar{H}e^{-iG}\\ \; & =\sum_{\left(iQ_{\|}\right)}E_{\left(iQ_{\|}\right)}X_{\left(iQ_{\|}\right)}^{†}X_{\left(iQ_{\|}\right)}+\omega_{\sigma_{+}}c_{\sigma_{+}}^{†}c_{\sigma_{+}}\\ \; & +M_{\sigma_{+}}\left[c_{\sigma_{+}}^{†}X_{\left(1\Gamma \right)}+c_{\sigma_{+}}X_{\left(1\Gamma \right)}^{†}\right]+\sum_{\alpha q_{\|}}\Omega_{\alpha ,q_{\|}}\left(b_{\alpha ,q_{\|}}^{†}b_{\alpha ,q_{\|}}-{\tilde{b}}_{\alpha ,q_{\|}}^{†}{\tilde{b}}_{\alpha ,q_{\|}}\right)\\ \; & +\sum_{ij\alpha Q_{\|}q_{\|}}D_{\alpha q_{\|}}^{ij}X_{\left(jQ_{\|}+q_{\|}\right)}^{†}X_{\left(iQ_{\|}\right)}\left[\cosh\left(\theta_{\alpha ,q_{\|}}\right)\left(b_{\alpha ,q_{\|}}+b_{\alpha ,-q_{\|}}^{†}\right)+\sinh\left(\theta_{\alpha ,q_{\|}}\right)\left({\tilde{b}}_{\alpha ,-q_{\|}}+{\tilde{b}}_{\alpha ,q_{\|}}^{†}\right)\right] \end{array}$$

in which

(7) $$\begin{array}{c} G=G^{†}=-i\sum_{\alpha q_{\|}}\theta_{\alpha ,q_{\|}}\left(b_{\alpha ,q_{\|}}{\tilde{b}}_{\alpha ,q_{\|}}-b_{\alpha ,q_{\|}}^{†}{\tilde{b}}_{\alpha ,q_{\|}}^{†}\right) \end{array}$$

is the Bogoliubov operator, and

(8) $$\begin{array}{c} \theta_{\alpha ,q_{\|}}=\mathrm{arctanh}\left(e^{-\beta \Omega_{\alpha ,q_{\|}}/2}\right) \end{array}$$

are the mixing angles, which account for the influence of the temperature on the exciton–phonon couplings.

The Hamiltonian of Equation (6) commutes with the number operator

(9) $$N_{\mathrm{ex}}=c_{\sigma_{+}}^{†}c_{\sigma_{+}}+\sum_{\left(iQ_{\|}\right)}X_{\left(iQ_{\|}\right)}^{†}X_{\left(iQ_{\|}\right)}$$

and conserves the number of excitons. In this work, we consider the dynamics within the singly excited excitonic manifold, where $\langle N_{\mathrm{ex}}\rangle =1$. In this manifold, the solution of the time-dependent Schrödinger equation with the TFD Hamiltonian of Equation (6) can be represented in terms of the mDA wave function of multiplicity *M*:

(10) $$|D_{2}^{M}\left(t\right)\rangle =\sum_{i=0}|i\rangle \sum_{m=1}^{M}B_{mi}\left(t\right)e^{\sum_{l}\left(u_{ml}\left(t\right)b_{l}^{†}-H.C.\right)}|0\rangle \times e^{\sum_{q}\left({\tilde{u}}_{mq}\left(t\right){\tilde{b}}_{q}^{†}-H.C.\right)}|\tilde{0}\rangle$$

Here, the indices i=0,1,2,3 denote the photon state, KK, KQ, and ${\mathrm{KK}}^{\prime }$ excitons, respectively. $B_{mi}\left(t\right)$ represents the time-dependent exciton amplitude, where the indices *m* stand for the *m*th coherent state superposition (with a total of M superpositions). The operators $b_{l}^{†}\left(b_{l}\right)$ and ${\tilde{b}}_{l}^{†}\left(b_{l}\right)$ are the physical and tilde phonon’s creation (annihilation) operators, respectively. The variables $u_{ml}\left(t\right)$ and ${\tilde{u}}_{ml}\left(t\right)$ capture displacements of the physical and tilde phonons in *m*th coherent state. For the calculation of exciton dynamics, the initial population is solely in the photon state, and the initial elements of $u_{ml}\left(t\right)$ and ${\tilde{u}}_{ml}\left(t\right)$ are random numbers of the order $10^{-4}$. The parameters $B_{mi}\left(t\right)$, $u_{ml}\left(t\right)$, and ${\tilde{u}}_{ml}\left(t\right)$ are determined through the equations of motion obtained following the variational principle [41,46,64], as detailed in Appendix A. All observables studied in the present work were evaluated in $|D_{2}^{M}\left(t\right)\rangle$, as described in Appendix B.

To establish a realistic model, ab initio input parameters entering the Hamiltonian include the electronic band structure [65], phonon dispersion [56], dielectric constants [66] and electron–phonon coupling elements [56]. In our calculations, we assumed that $E_{\left(1\Gamma \right)}=1.724\;\mathrm{eV}$ [67,68], $E_{\left(2\Lambda \right)}=1.69\;\mathrm{eV}$, and $E_{\left(3K\right)}=1.678\;\mathrm{eV}$, where the spectral exciton separations between KK, KQ, and ${\mathrm{KK}}^{\prime }$ excitons were taken from ref. [5]. The exciton energies and wave functions were derived in the effective mass approximation by solving the Wannier equation [69,70,71]. This approach leads to an exciton dispersion, which is comparable to that from DFT calculations based on the Bethe–Salpeter equation [72,73,74]. To amplify cavity-prompted phenomena, a single cavity mode [5,75] was taken to be in resonance with the luminescent KK exciton, where $\hbar \omega_{\sigma_{+}}=1.724\;\mathrm{eV}$. The dielectric constant [5,66] was set to be 4.5 for the hBN-encapsulated TMDs monolayers. For the exciton–cavity couplings, we only consider the interactions between the bright KK exciton and photons, as there is no transition dipole moment of momentum–dark states. Within the photonic crystal cavities, the coupling between excitons and photons is modifiable within a range from 4meV to 14meV, as documented by Rosser et al. [31]; for our calculations, $M_{\sigma_{+}}=0,4,8,12\mathrm{meV}$ [76,77,78] were used. Table 1 lists the employed phonon frequencies [5,56], illustrating that both longitudinal acoustic (LA) and transverse acoustic (TA) phonon branches exhibit a linear dispersion nearing the long-wavelength limit, aligning their frequencies at zero when q=0. The optical phonon branches with significant exciton interactions include the homopolar ($A_{1}$) modes, characterized by out-of-plane vibrations, and the in-plane longitudinal (LO) and transversal (TO) modes.

The Hamiltonian of Equation (6) incorporates 23 phonon modes, three excitonic states (KK, KQ, ${\mathrm{KK}}^{\prime }$), and a single photonic mode. The number of physical phonon modes (i.e., 23) surpasses 13, as outlined in Table 1, which is due to the inclusion of ± modes $b_{\alpha ,\pm q_{\|}}$ with $\alpha =\mathrm{KQ},{\mathrm{KK}}^{\prime }$. The total number of phonon modes (i.e., 46) comes about by the duplication of the number of physical phonons owing to the inclusion of the tilde modes ${\tilde{b}}_{\alpha ,q_{\|}}$, crucial for addressing temperature effects within the TFD framework. The model omits holes near the K point due to their ${\mathrm{KK}}^{\prime }$ symmetry and excludes spin-forbidden dark states, which are irrelevant for the ultrafast dynamics under study. This model effectively captures coherent interactions among excitons, phonons, and photons, and it incorporates environmental dephasing as proposed, e.g., in Ref. [5].

The impact of the temperature on the exciton dynamics and TRF spectra has been comprehensively investigated in Ref. [55]. Here, we set the temperature to 75 K and focused on a detailed study of how the strength of the EC coupling affects the dynamical and spectroscopic observables. All our numerical results are proven to be convergent for the multiplicity M=48 of the mDA wave function of Equation (10).

## 3. Results and Discussion

### 3.1. Exciton Populations

**Figure 2:**
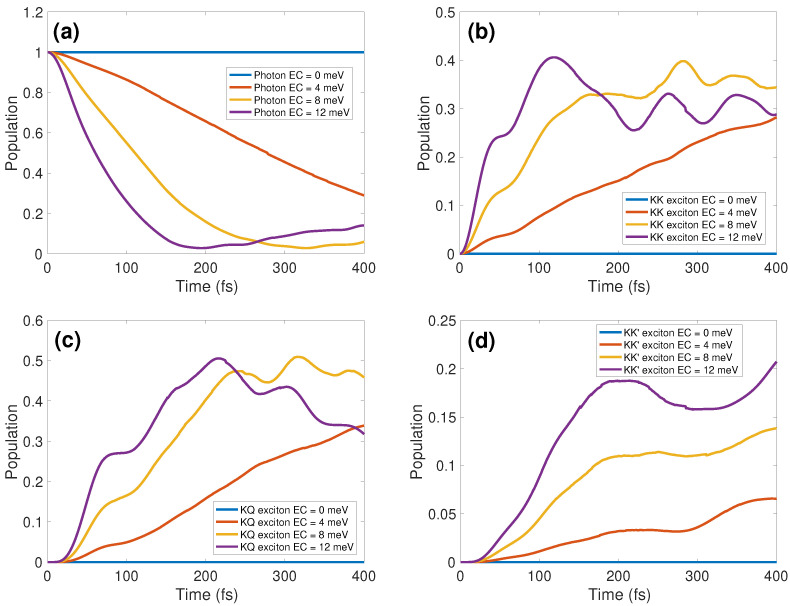
Population plots of (**a**) photons, (**b**) KK excitons, (**c**) KQ excitons, and (**d**) KK’ excitons for different EC coupling strengths.

Figure 2 provides a comprehensive analysis of how varying ECs influence the dynamics of specific photonic and excitonic modes. In the absence of EC coupling in panel (a), the photon mode population persists, reflecting the lack of energy exchange between photons and excitons. For EC=4 meV (red lines), the photon mode population decreases, and the excitonic mode population increases almost linearly with time, exhibiting the short-time ballistic transport on the timescale of ∼400 fs. As the EC coupling strength increases, additional oscillations and nonmonotonic behaviors emerge, as depicted in panels (b)–(d). This is indicative of exciton–polariton formation resulting from intensified exciton–photon interactions within the cavity. It is the value of EC=8 meV (yellow lines) that marks the changeover to the oscillatory strong EC coupling regime. The onset of this regime is manifested through the nonmonotonic evolution of the cavity mode population in panel (a), which reaches a minimum (almost zero) at t≈350 fs (when EC=8 meV) and t≈200 fs (when EC=12 meV) followed by the increase at longer times. Irrespective of the EC coupling strength, the excitonic states are populated sequentially [55] at short times: the KK state is populated first, followed by the KQ and KK’ states. Enhanced exciton–cavity coupling accelerates the transfer of photon population to excitons, as evidenced by the purple lines for EC=12 meV, reaching their minima (a) and maxima (b)–(d) more swiftly. At an EC of 12 meV both bright KK and dark KQ excitons acquire rapid population growth initially at 100 fs and 200 fs, respectively, followed by noticeable oscillations. Remarkably, an increased EC consistently sustains a higher (though nonmonotonic as a function of time) population level in the dark KK’ exciton due to phonon-assisted transfer when the polaritonic state is formed. This observation, which demonstrates robustness of the cavity-induced population of the KK’ excitons within a wide range of EC coupling strengths, may become important for practical applications.

### 3.2. TRF

To delve deeper into the dynamics of ultrafast exciton–polariton formation in WSe_2_ monolayers, we computed the TRF spectra S(ω,t) presuming instantaneous excitation of the system by the pump pulse. These spectra, which describe the rate of emission of photons of frequency ω at time *t* [64,79,80], are evaluated with the mDA wave function, as described in Appendix B. For obtaining the spectral shape of S(ω,t), we used the overdamped harmonic oscillator lineshape function of Equation (A9) with the Stokes shift parameter λ=5 meV and the memory rate parameter Λ=30 meV.

**Figure 3:**
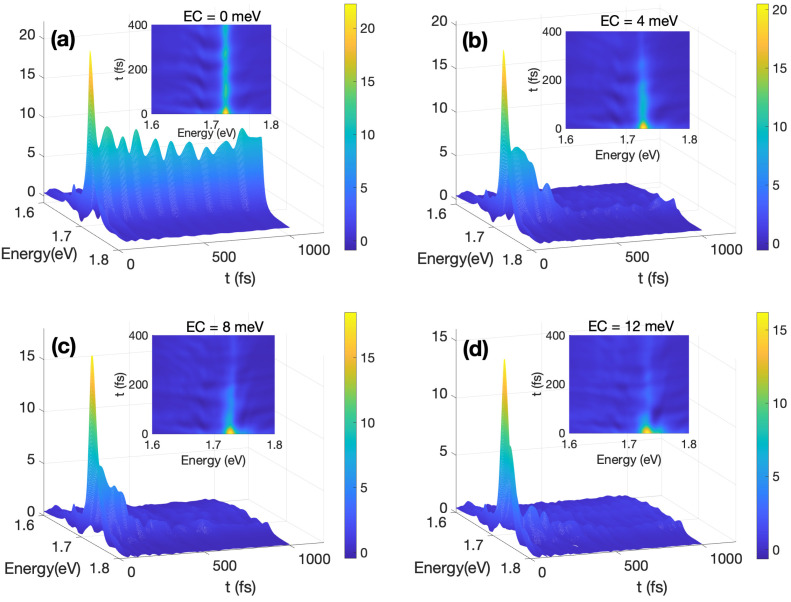
A 3D view of TRF spectra S(ω,t) within the first 1000 fs: (**a**) EC=0 meV, (**b**) EC=4 meV, (**c**) EC=8 meV, and (**d**) EC=12 meV. Inset figures are 2D view of TRF spectra S(ω,t) within the first 400 fs.

A 3D view of the TRF spectra S(ω,t) is presented in Figure 3 for different ECs from zero (a) through 4 eV (b) and 8 eV (c) to 12 eV (d). The spectra are characterized by a high initial (at t=0) peak revealing the bright KK exciton (direct PL), the intensity of which drops substantially on the timescale of several dozens femtoseconds. Rapid fluorescence attenuation of the bright KK exciton is culminated in panel (d), which is characterized by an approximately 90% loss in intensity within the initial 100 fs. This behavior is indicative of swift internal conversion processes at the (cavity-induced) CIs [45,46,81]. In contrast, the absence of a cavity (EC=0, panel (a)) sees the fluorescence intensity of direct PL at about 50% in the first 100 fs. From panels (a) to (d), the tendency of fast fluorescence decay with the EC coupling agrees with the enhanced population of the dark states (KQ and KK’) indicated in Figure 2c,d.

In the time domain, S(ω,t) exhibits oscillatory behavior, which mirrors cavity-mediated exciton–phonon wave packet motion. This motion is especially pronounced for EC=0 (panel a). However, it is not strictly periodic, since the spectral maxima are separated by the intermittent time interval of about ∼80–100 fs. Interestingly, these intervals are substantially shorter than the period corresponding to the fastest phonon mode ($2\pi /\Omega_{LO,\Lambda }=127$ fs. This is a clear indication of the exciton–vibrational–photon coupling, which results in Rabi-like oscillations. As EC coupling strengths increase, the wave packet evolution retains oscillatory features, which, however, become progressively more erratic and spread over a wider range in the frequency domain.

A detailed examination of the early stages of the spectral evolution is provided by the inset of Figure 3, which elucidates the wave packet dynamics influenced by the intrinsic and cavity-induced CIs. The spectral features primarily cluster around ω=1.724 eV, aligning with the bright KK exciton energy. Notably, this observation confirms that the lower-energy KQ and ${\mathrm{KK}}^{\prime }$ excitons, which are intrinsically dark, do not substantially contribute to the emission at these early times (cf. Ref. [82]). This scenario also illustrates the influence of polaritonic effects in Figure 3d: the fundamental excitonic KK state splits into two distinct polaritonic states, with a separation roughly equal to $2M_{\sigma_{-}}=24\;\mathrm{meV}$, thereby widening the spectrum. Additionally, interactions with phonon modes (polaron effects) further broaden the spectrum, extending it toward the blue side. Inversely, decreasing ECs causes a narrowing of S(ω,t) along the ω axis, which produces a more symmetric spectral shape and leads to a slower fluorescence quenching.

**Figure 4:**
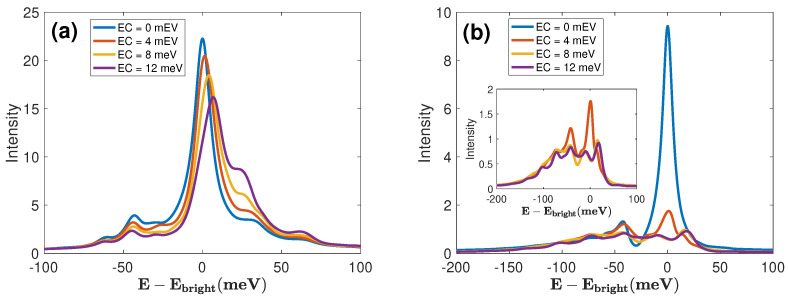
TRF spectra S(ω,t) at t=0 (**a**) and 1000 fs (**b**) for four EC coupling strengths, as indicated in the panels.

For obtaining a detailed view of the spectral features, it is worthwhile to inspect S(ω,t) at specific values of *t*. TRF spectra at t=0, which can be interpreted as absorption spectra [79], are shown in panel (a) of Figure 4, accompanied by relaxed fluorescence spectra at t=1000 fs in panel (b). At t=0, the spectra for all EC couplings exhibit similar shapes. Yet, larger EC coupling strengths reduce the intensity of the main peak associated with direct PL, as well as inducing a blue shift and spectral broadening. The red wing peak at $\omega \approx E-E_{\mathrm{bright}}=-43$ meV in Figure 4a is related to indirect PL, while the blue wing shoulder located around $\omega \approx E-E_{\mathrm{bright}}=30$ meV represents a vibronic peak. This implies a modification (enhancement) of the Huang–Rhys factor [83], and a similar spectral redistribution is observed in surface plasmon polaritons [84]. The observed enhancement of the vibronic peak intensity, as outlined in Ref. [85], stems from the close-to-resonance conditions. If the phonon frequency $\Omega_{\alpha ,q_{\|}}$ (25–30 meV) from the energy gap between the prominent KK peak and the adjacent higher energy peak matches the separation Δ between the red wing peak and the central peak, the resonance condition is satisfied. This relationship is quantified as $\Delta \approx k\Omega_{\alpha ,q_{\|}}$, where k=1,2,.... As EC couplings increase from 0 to 12 meV, Δ nearly doubles the frequency of the (Λ and K) phonon modes.

The relaxed TRF spectrum at t=1000 fs in Figure 4b shows drastic changes in lineshape as a function of the EC coupling strength. The intensity of the central direct PL peak (EC = 0) diminishes significantly due to the EC-enhanced population transfer to the dark excitons. In the strong coupling regime (EC=8 meV and EC=12 meV), the peak splits into two sub-peaks separated by 25 meV, as shown in the inset. These polaritonic features are not seen in the absorption spectrum. Therefore, TRF spectra deliver valuable information on the photoinduced processes in WSe_2_ monolayers, which cannot be extracted from the absorption spectra.

**Figure 5:**
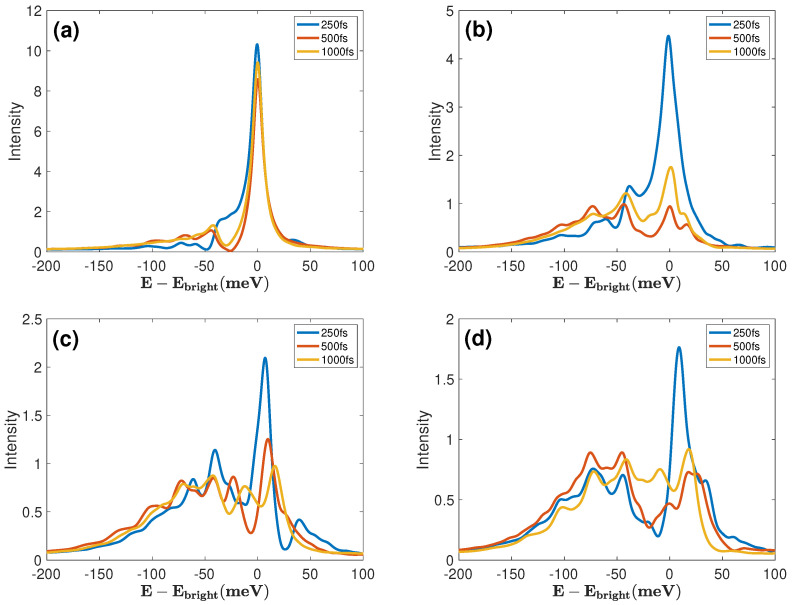
TRF spectra S(ω,t) at 250 fs, 500 fs, and 1000 fs for (**a**) EC=0 meV, (**b**) EC=4 meV, (**c**) EC=8 meV, and (**d**) EC=12 meV.

Figure 5 presents the TRF spectra S(ω,t) at intermediate times for four EC coupling strengths. For EC=0 [panel (a)], the main dynamical events in the evolution of S(ω,t) are over before t=250 fs (see Figure 3a). Hence, the spectra at 500 fs and 1000 fs in Figure 5a are almost identical, suggesting that the steady state fluorescence is reached quicker for weaker EC coupling. Only the intensity of the KK peak goes out slowly and remains much higher than that of the adjacent vibronic peaks. Additionally, the KK peak position stays unchanged in panels (a) and (b), contrasting with the significant shifts of the peak positions in panels (c) and (d). These shifts can be attributed to the polaritonic effects, which are manifested in significantly richer peak structures that spread over a substantially broader spectral domain. This indicates that the widths of relaxed TFR spectra can be used for estimating the EC coupling strengths. Qualitatively, the spectra in the strong EC coupling regime (panel d) are similar to those calculated in Ref. [31] using the Lindblad master equation for cavity-assisted TMDs monolayers. Nonetheless, the inter-peak separations and spectral widths in our spectra are significantly larger than those in Ref. [31] due to the stronger exciton–phonon and exciton–photon interactions.

## 4. Conclusions

By integrating the mDA method [41,42] with the TFD framework [49,50], we performed numerically accurate simulations of the exciton dynamics and TRF spectra of cavity-modulated WSe_2_ monolayers at 75 K for EC coupling strengths ranging from 0 (no polaritonic effects) to 12 meV (strong polaritonic effects). The efficient computational method employed in the present work can be applied to simulations of various cavity-tuned 2D materials at finite temperatures. More specifically, our approach can be extended to possibly account for the inversion of the band ordering (i.e., the lower polariton mode pushed below the WSe_2_ dark state) and phonon-assisted polariton relaxation [86]. Furthermore, anti-Stokes PL [87] activated in nanocavity-integrated WSe_2_ monolayers can be simulated by incorporating resonant excitation of a dark exciton [87] in our Hamiltonian.

Our approach identified distinct spectroscopic signatures and characteristic timescales of polaronic and polaritonic effects in WSe_2_ monolayers at various EC interactions. Notably, our investigation highlighted the crucial role of multidimensional CIs in governing the dynamics of strongly coupled excitons, photons, and phonons. It comes as no surprise that the EC coupling is an important parameter governing energy transfer in TMDs. What is more significant for applications and less evident is that we found that several aspects of the polaritonic transport in WSe_2_ monolayers do not require finetuning of EC interactions. For example, increasing EC coupling strengths enhances the total population of the dark interval excitons KQ and KK’ on the timescale of up to 200 fs, and it amplifies populations of the KK’ excitons at times longer than 400 fs. This robustness in the cavity-mediated energy transport may be instrumental for engineering 2D materials.

## Figures and Tables

**Table 1:** Phonon frequencies [56] (in units of meV).

Mode	Γ	Λ	*K*
TA	0	11.6	15.6
LA	0	14.3	18
TO	30.5	27.3	26.7
LO	30.8	32.5	31.5
$A_{1}$	30.8	30.4	31

## Data Availability

The data presented in this study are available upon request from the corresponding author.

## Supplementary Materials

The following supporting information can be downloaded at: https://www.mdpi.com/article/10.3390/ma17164127/s1.

## Abbreviations

The following abbreviations are used in this manuscript:

TMDs	Transition metal dichalcogenides
2D	Two-dimensional
PL	Photoluminescence
TRF	Time-resolved fluorescence
mDA	Multiple Davydov Ansatz
CIs	Conical intersections
TFDs	Thermofield dynamics
EC	Exciton–cavity

## Appendix A. Equations of Motion for the mDA Parameters

The time evolution of the parameters $B_{mi}\left(t\right)$, $u_{ml}\left(t\right)$, and ${\tilde{u}}_{mq}\left(t\right)$ specifying the mDA wave function (10) are determined using the variational method [41,46,64]:

(A1) $$\begin{array}{c} \frac{d}{dt}\frac{\partial L}{\partial {\dot{\xi }}_{j}^{*}}-\frac{\partial L}{\partial \xi_{j}^{*}}=0, \end{array}$$

where the Lagrangian L is defined as

(A2) $$\begin{array}{ccc} L & = & \frac{i}{2}\left[\langle D_{2}^{M}\left(t\right)|{\dot{D}}_{2}^{M}\left(t\right)\rangle -\langle {\dot{D}}_{2}^{M}\left(t\right)|D_{2}^{M}\left(t\right)\rangle \right]-\langle D_{2}^{M}\left(t\right)|H_{T}|D_{2}^{M}\left(t\right)\rangle . \end{array}$$

The multi-$D_{2}$ Ansatz converges to reproduce the accurate wave function if the multiplicity *M* is large enough. The multiplicity M=48 adopted in our simulations yields the converged results.

The Hamiltonian structured within the framework of the multi-$D_{2}$ ansatz is expressed as

(A3) $$\begin{array}{cc} \; & L_{H}=\langle D_{2}^{M}\left(t\right)|{\bar{H}}_{\theta }|D_{2}^{M}\left(t\right)\rangle \\ \; & =\sum_{i=0}^{3}\sum_{m}^{M}\sum_{m^{\prime }}^{M}E_{i}B_{mi}^{*}B_{m^{\prime }i}R_{mm^{\prime }}+\sum_{i=0}^{3}\sum_{m}^{M}\sum_{m^{\prime }}^{M}B_{mi}^{*}B_{m^{\prime }i}\sum_{l}^{23}\Omega_{l}\left(u_{ml}^{*}u_{m^{\prime }l}-{\tilde{u}}_{ml}^{*}{\tilde{u}}_{m^{\prime }l}\right)R_{mm^{\prime }}\\ \; & +\sum_{m}^{M}\sum_{m^{\prime }}^{M}\left(M_{\sigma_{+}}B_{m0}^{*}B_{m^{\prime }1}+M_{\sigma_{+}}^{*}B_{m1}^{*}B_{m^{\prime }0}\right)R_{mm^{\prime }}+\sum_{m}^{M}\sum_{m^{\prime }}^{M}\{\sum_{l=1}^{3}D_{1l}^{\left(1\right)}B_{m1}^{*}B_{m^{\prime }1}\cosh\left(\theta_{l}\right)\left(u_{ml}^{*}+u_{m^{\prime }l}\right)R_{mm^{\prime }}\\ \; & +\sum_{l=1}^{3}D_{1l}^{\left(1\right)}B_{m1}^{*}B_{m^{\prime }1}\sinh\left(\theta_{l}\right)\left({\tilde{u}}_{ml}^{*}+{\tilde{u}}_{m^{\prime }l}\right)R_{mm^{\prime }}+\sum_{l=4}^{8}D_{l}^{\left(2\right)}B_{m1}^{*}B_{m^{\prime }2}\left(u_{ml}^{*}\cosh\left(\theta_{l}\right)+{\tilde{u}}_{m^{\prime }l}\sinh\left(\theta_{l}\right)\right)R_{mm^{\prime }}\\ \; & +\sum_{l=14}^{18}D_{l}^{\left(2\right)}B_{m1}^{*}B_{m^{\prime }2}\left(u_{m^{\prime }l}\cosh\left(\theta_{l}\right)+{\tilde{u}}_{ml}^{*}\sinh\left(\theta_{l}\right)\right)R_{mm^{\prime }}\\ \; & +\sum_{l=9}^{13}D_{l}^{\left(3\right)}B_{m1}^{*}B_{m^{\prime }3}\left(u_{ml}^{*}\cosh\left(\theta_{l}\right)+{\tilde{u}}_{m^{\prime }l}\sinh\left(\theta_{l}\right)\right)R_{mm^{\prime }}\\ \; & +\sum_{l=19}^{23}D_{l}^{\left(3\right)}B_{m1}^{*}B_{m^{\prime }3}\left(u_{m^{\prime }l}\cosh\left(\theta_{l}\right)+{\tilde{u}}_{ml}^{*}\sinh\left(\theta_{l}\right)\right)R_{mm^{\prime }}\}\\ \; & +\sum_{m}^{M}\sum_{m^{\prime }}^{M}\{\sum_{l=1}^{3}D_{2l}^{\left(1\right)}B_{m2}^{*}B_{m^{\prime }2}\cosh\left(\theta_{l}\right)\left(u_{ml}^{*}+u_{m^{\prime }l}\right)R_{mm^{\prime }}+\sum_{l=1}^{3}D_{2l}^{\left(1\right)}B_{m2}^{*}B_{m^{\prime }2}\sinh\left(\theta_{l}\right)\left({\tilde{u}}_{ml}^{*}+{\tilde{u}}_{m^{\prime }l}\right)R_{mm^{\prime }}\\ \; & +\sum_{l=4}^{8}D_{l}^{\left(2\right)}B_{m2}^{*}B_{m^{\prime }1}\left(u_{ml}^{*}\cosh\left(\theta_{l}\right)+{\tilde{u}}_{m^{\prime }l}\sinh\left(\theta_{l}\right)\right)R_{mm^{\prime }}\\ \; & +\sum_{l=14}^{18}D_{l}^{\left(2\right)}B_{m2}^{*}B_{m^{\prime }1}\left(u_{m^{\prime }l}\cosh\left(\theta_{l}\right)+{\tilde{u}}_{ml}^{*}\sinh\left(\theta_{l}\right)\right)R_{mm^{\prime }}\}\\ \; & +\sum_{m}^{M}\sum_{m^{\prime }}^{M}\{\sum_{l=1}^{3}D_{3l}^{\left(1\right)}B_{m3}^{*}B_{m^{\prime }3}\cosh\left(\theta_{l}\right)\left(u_{ml}^{*}+u_{m^{\prime }l}\right)R_{mm^{\prime }}+\sum_{l=1}^{3}D_{3l}^{\left(1\right)}B_{m3}^{*}B_{m^{\prime }3}\sinh\left(\theta_{l}\right)\left({\tilde{u}}_{ml}^{*}+{\tilde{u}}_{m^{\prime }l}\right)R_{mm^{\prime }}\\ \; & +\sum_{l=9}^{13}D_{l}^{\left(3\right)}B_{m3}^{*}B_{m^{\prime }1}\left(u_{ml}^{*}\cosh\left(\theta_{l}\right)+{\tilde{u}}_{m^{\prime }l}\sinh\left(\theta_{l}\right)\right)R_{mm^{\prime }}\\ \; & +\sum_{l=19}^{23}D_{l}^{\left(3\right)}B_{m3}^{*}B_{m^{\prime }1}\left(u_{m^{\prime }l}\cosh\left(\theta_{l}\right)+{\tilde{u}}_{ml}^{*}\sinh\left(\theta_{l}\right)\right)R_{mm^{\prime }} \end{array}$$

Here, $D_{il}^{\left(1\right)}$ specifies the diagonal exciton–phonon coupling matrix elements for the exciton state |i〉 interacting with phonon mode *l*. The terms $D_{l}^{\left(2\right)}$ and $D_{l}^{\left(3\right)}$ represent the exciton–phonon couplings between KK exciton states and KQ (${\mathrm{KK}}^{\prime }$) exciton states with phonon mode *l*, respectively. Consequently, the governing equations for $B_{m^{\prime }0}$ are formulated as $B_{m^{\prime }1}$, $B_{m^{\prime }2}$, and $B_{m^{\prime }3}$, which are formulated as

(A4) $$\begin{array}{cc} \; & i\sum_{m^{\prime }}^{M}\left[{\dot{B}}_{m^{\prime }0}+B_{m^{\prime }0}\sum_{l}u_{ml}^{*}{\dot{u}}_{m^{\prime }l}+B_{m^{\prime }0}\sum_{l}{\tilde{u}}_{ml}^{*}{\tilde{\dot{u}}}_{m^{\prime }l}\right]R_{mm^{\prime }}\\ \; & =\sum_{m^{\prime }}^{M}E_{0}B_{m^{\prime }0}R_{mm^{\prime }}+\sum_{m^{\prime }}^{M}B_{m^{\prime }0}\sum_{l}^{23}\Omega_{l}\left(u_{ml}^{*}u_{m^{\prime }l}-{\tilde{u}}_{ml}^{*}{\tilde{u}}_{m^{\prime }l}\right)R_{mm^{\prime }}\\ \; & +\sum_{m^{\prime }}^{M}M_{\sigma_{+}}B_{m^{\prime }1}R_{mm^{\prime }} \end{array}$$

Other variational parameters, such as $B_{m^{\prime }1}$, $B_{m^{\prime }2}$, $B_{m^{\prime }3}$, $u_{m^{\prime }l}$, and ${\tilde{u}}_{m^{\prime }l}$, are described in the Supplementary Materials.

## Appendix B. Observables in Terms of Multi-D_2_ Wave Functions

Populations of the photonic and excitonic states can be evaluated in terms of the multi-_2_ wave functions (10) as the expectation values of the projection operators:

(A5) $$P_{i}\left(t\right)=\langle D_{2}^{M}\left(t\right)|i\rangle \langle i|D_{2}^{M}\left(t\right)\rangle$$

where i=0,1,2,3 correspond to the photon state, KK, KQ, and KK′ excitons, respectively.

TRF spectra excited by a short (Dirac delta function) pump pulse can be computed using the following expression [64,79,80]:

(A6) S(ω,t)∼Re∫0∞dt3R1DA(t3,t,0)R1g(t)(t3,t,0)exp[iωt3].

Here, $R_{1}^{DA}\left(t_{3},t,0\right)$ is the third-order response function evaluated using the mDA approach as follows:

(A7) $$\begin{array}{cc} R_{1}^{DA}\left(t_{3},t_{2},t_{1}\right)= & \sum_{m,m^{\prime }}^{M}\sum_{i_{0},i_{1},i_{2},i_{3}}^{3}\mu_{i_{2}}^{*}\mu_{i_{3}}\mu_{i_{0}}^{*}\mu_{i_{1}}B_{m^{\prime }i_{1}i_{0}}^{*}\left(t_{2}\right)B_{mi_{2}i_{3}}\left(t_{3}+t_{2}+t_{1}\right)\\ \; & \times e^{\sum_{q}u_{m^{\prime }qi_{0}}^{*}\left(t_{2}\right)u_{mqi_{3}}\left(t_{3}+t_{2}+t_{1}\right)e^{i\Omega_{q}t}+\sum_{l}{\tilde{u}}_{m^{\prime }li_{0}}^{*}\left(t_{2}\right){\tilde{u}}_{mli_{3}}\left(t_{3}+t_{2}+t_{1}\right)e^{i\Omega_{l}t}}\\ \; & \times e^{-\frac{1}{2}\sum_{q}\left(\right|u_{m^{\prime }qi_{0}}\left(t_{2}\right){|}^{2}+|u_{mqi_{3}}\left(t_{3}+t_{2}+t_{1}\right){|}^{2})-\frac{1}{2}\sum_{l}\left(\right|{\tilde{u}}_{m^{\prime }li_{0}}\left(t_{2}\right){|}^{2}+|{\tilde{u}}_{mli_{3}}\left(t_{3}+t_{2}+t_{1}\right){|}^{2})} \end{array}$$

Here $\mu_{i}$ are the transition dipole moment coupling coefficients, where $\mu_{0}$ = $\mu_{1}$ = 1, $\mu_{2}$ = $\mu_{3}$ = 0. $B_{m^{\prime }i_{1}i_{0}}\left(t\right)$ indicates the probability at time *t* for the exciton to occupy the state $|i_{1}\rangle$ relative to the initial state $|i_{0}\rangle$ with a given multiplicity *M*, while $u_{m^{\prime }qi_{0}}\left(t\right)$ and ${\tilde{u}}_{m^{\prime }li_{0}}\left(t\right)$ denote displacements of the ‘physical’ and ‘tilde’ phonon modes.

$R_{1}^{g(t)}\left(t_{3},t,0\right)$ is the response function representing environmental factors, which are not included into the Hamiltonian of Equation (6). It assumes the form

(A8) $$\begin{array}{c} R_{1}^{g(t)}\left(t_{3},t_{2},t_{1}\right)=\exp\left[-g\left(t_{1}\right)-g^{*}\left(t_{2}\right)-g^{*}\left(t_{3}\right)+g\left(t_{1}+t_{2}\right)+g^{*}\left(t_{2}+t_{3}\right)-g\left(t_{1}+t_{2}+t_{3}\right)\right] \end{array}$$

and can be computed using the overdamped harmonic oscillator lineshape functions [64,79]:

(A9) $$\begin{array}{cc} \; & g\left(t\right)=g^{\prime }\left(t\right)+ig^{\prime \prime }\left(t\right) \end{array}$$

(A10) $$g^{\prime \prime }\left(t\right)=-\left(\lambda /\Lambda \right)\left[\exp\left(-\Lambda t\right)+\Lambda t-1\right]$$

(A11) $$\begin{array}{c} g^{\prime }\left(t\right)=\left(\lambda /\Lambda \right)\cot\left(\hbar \beta \Lambda /2\right)\left[\exp\left(-\Lambda t\right)+\Lambda t-1\right]\\ +\frac{4\lambda \Lambda }{\hbar \beta }\sum_{n=1}^{\infty }\frac{\exp\left(-\upsilon_{n}t\right)+\upsilon_{n}t-1}{\upsilon_{n}\left({\upsilon_{n}}^{2}-\Lambda^{2}\right)} \end{array}$$

(A12) $$\begin{array}{cc} \; & \upsilon_{n}=\frac{2\pi }{\hbar \beta }n. \end{array}$$

Here, λ is the Stokes shift, and $\Lambda^{-1}$ represents the memory time of the environment.
