# Supplementary material for: Cavity-Tuned Exciton Dynamics in Transition Metal Dichalcogenides Monolayers
Source: Materials (Basel). 2024 Aug 20;17(16):4127. doi: 10.3390/ma17164127 (PMC11356741; doi:10.3390/ma17164127)
Supplement: Supplementary file 1 [file materials-17-04127-s001.zip › Supplementary Materials.pdf]

Supplementary Materials:

Cavity-Tuned Exciton Dynamics in Transition-Metal Dichalcogenides Monolayers

Kaijun Shen,[†] Kewei Sun,[‡] Maxim F. Gelin,[‡] and Yang Zhao[*,†]

†*School of Materials Science and Engineering, Nanyang Technological University, Singapore 639798, Singapore*

‡*School of Science, Hangzhou Dianzi University, Hangzhou 310018, China*

E-mail: YZhao@ntu.edu.sg

S1. Equations of motion for variational parameters

Other variational parameters, such as $B_{m'1}$, $B_{m'2}$, $B_{m'3}$ are given by

$$
\begin{aligned}
& i\sum_{m'}^{M}[\dot{B}_{m'1} + B_{m'1}\sum_{l} u^*_{ml}\dot{u}_{m'l} + B_{m'1}\sum_{l} \tilde{u}^*_{ml}\dot{\tilde{u}}_{m'l}]R_{mm'} \\
& = \sum_{m'}^{M} E_1 B_{m'1}R_{mm'} + \sum_{m'}^{M} B_{m'1}\sum_{l}^{23}\Omega_l(u^*_{ml}u_{m'l} - \tilde{u}^*_{ml}\tilde{u}_{m'l})R_{mm'} \\
& + \sum_{m'}^{M} M^*_{\sigma_+}B_{m'0}R_{mm'} + \sum_{m'}^{M}\{\sum_{l=1}^{3} D^{(1)}_{1l}B_{m'1}\cosh(\theta_l)(u^*_{ml} + u_{m'l})R_{mm'} \\
& + \sum_{l=1}^{3} D^{(1)}_{1l}B_{m'1}\sinh(\theta_l)(\tilde{u}^*_{ml} + \tilde{u}_{m'l})R_{mm'} \\
& + \sum_{l=4}^{8} D^{(2)}_{l}B_{m'2}(u^*_{ml}\cosh(\theta_l) + \tilde{u}_{m'l}\sinh(\theta_l))R_{mm'} + \sum_{l=14}^{18} D^{(2)}_{l}B_{m'2}(u_{m'l}\cosh(\theta_l) + \tilde{u}^*_{ml}\sinh(\theta_l))R_{mm'} \\
& + \sum_{l=9}^{13} D^{(3)}_{l}B_{m'3}(u^*_{ml}\cosh(\theta_l) + \tilde{u}_{m'l}\sinh(\theta_l))R_{mm'} + \sum_{l=19}^{23} D^{(3)}_{l}B_{m'3}(u_{m'l}\cosh(\theta_l) + \tilde{u}^*_{ml}\sinh(\theta_l))R_{mm'}\}
\end{aligned}
\tag{S1}
$$

$$
\begin{aligned}
& i\sum_{m'}^{M}[\dot{B}_{m'2} + B_{m'2}\sum_{l} u^*_{ml}\dot{u}_{m'l} + B_{m'2}\sum_{l} \tilde{u}^*_{ml}\dot{\tilde{u}}_{m'l}]R_{mm'} \\
& = \sum_{m'}^{M} E_2 B_{m'2}R_{mm'} + \sum_{m'}^{M} B_{m'2}\sum_{l}^{23}\Omega_l(u^*_{ml}u_{m'l} - \tilde{u}^*_{ml}\tilde{u}_{m'l})R_{mm'} \\
& + \sum_{m'}^{M}\{\sum_{l=1}^{3} D^{(1)}_{2l}B_{m'2}\cosh(\theta_l)(u^*_{ml} + u_{m'l})R_{mm'} + \sum_{l=1}^{3} D^{(1)}_{2l}B_{m'2}\sinh(\theta_l)(\tilde{u}^*_{ml} + \tilde{u}_{m'l})R_{mm'} \\
& + \sum_{l=4}^{8} D^{(2)}_{l}B_{m'1}(u^*_{ml}\cosh(\theta_l) + \tilde{u}_{m'l}\sinh(\theta_l))R_{mm'} + \sum_{l=14}^{18} D^{(2)}_{l}B_{m'1}(u_{m'l}\cosh(\theta_l) + \tilde{u}^*_{ml}\sinh(\theta_l))R_{mm'}\}
\end{aligned}
\tag{S2}
$$

$$
\begin{aligned}
& i\sum_{m'}^{M}[\dot{B}_{m'3} + B_{m'3}\sum_{l} u^{*}_{ml}\dot{u}_{m'l} + B_{m'3}\sum_{l}\tilde{u}^{*}_{ml}\dot{\tilde{u}}_{m'l}]R_{mm'} \\
& = \sum_{m'}^{M} E_3 B_{m'3}R_{mm'} + \sum_{m'}^{M} B_{m'3}\sum_{l}^{23}\Omega_l(u^{*}_{ml}u_{m'l} - \tilde{u}^{*}_{ml}\tilde{u}_{m'l})R_{mm'} \\
& + \sum_{m'}^{M}\{\sum_{l=1}^{3} D^{(1)}_{3l}B_{m'3}\text{cosh}(\theta_l)(u^{*}_{ml} + u_{m'l})R_{mm'} + \sum_{l=1}^{3} D^{(1)}_{3l}B_{m'3}\text{sinh}(\theta_l)(\tilde{u}^{*}_{ml} + \tilde{u}_{m'l})R_{mm'} \\
& + \sum_{l=9}^{13} D^{(3)}_{l}B_{m'1}(u^{*}_{ml}\text{cosh}(\theta_l) + \tilde{u}_{m'l}\text{sinh}(\theta_l))R_{mm'} + \sum_{l=19}^{23} D^{(3)}_{l}B_{m'1}(u_{m'l}\text{cosh}(\theta_l) + \tilde{u}^{*}_{ml}\text{sinh}(\theta_l))R_{mm'}\}
\end{aligned}
\tag{S3}
$$

Similarly, the equations of motion for $u_{m'l}$ and $\tilde{u}_{m'l}$are given by

$$
\begin{aligned}
& i\sum_{i=0}^{3}\sum_{m'}^{M} B^*_{mi}B_{m'i}\dot{u}_{m'l}R_{mm'} + i\sum_{i=0}^{3}\sum_{m'}^{M}[B^*_{mi}\dot{B}_{m'i} + B^*_{mi}B_{m'i}(\sum_{k} u^*_{mk}\dot{u}_{m'k} + \sum_{k}\tilde{u}^*_{mk}\dot{\tilde{u}}_{m'k})]R_{mm'}u_{m'l} \\
& = \sum_{i=0}^{3}\sum_{m'}^{M} E_iB^*_{mi}B_{m'i}R_{mm'}u_{m'l} + \sum_{i=0}^{3} B^*_{mi}B_{m'i}\Omega_l u_{m'l}R_{mm'} \\
& + \sum_{i=0}^{3}\sum_{m'}^{M} B^*_{mi}B_{m'i}\sum_{k}^{23}\Omega_k(u^*_{mk}u_{m'k} - \tilde{u}^*_{mk}\tilde{u}_{m'k})R_{mm'}u_{m'l} + \sum_{m'}^{M}\sum_{l} D^{(2)}_{l}B^*_{m2}B_{m'1}\cosh(\theta_l)R_{mm'}\delta_{l=4\sim 8} \\
& + \sum_{m'}^{M}(M_{\sigma_+}B^*_{m0}B_{m'1} + M^*_{\sigma_+}B^*_{m1}B_{m'0})R_{mm'}u_{m'l} + \sum_{m'}^{M}\sum_{i=1}^{3}\sum_{l=1}^{3} D^{(1)}_{il}B^*_{mi}B_{m'i}\cosh(\theta_l)R_{mm'}\delta_{l=1,2,3} \\
& + \sum_{m}^{M}\sum_{m'}^{M}\sum_{i=1}^{3}\sum_{k=1}^{3} D^{(1)}_{il}B^*_{mi}B_{m'i}\cosh(\theta_k)(u^*_{mk} + u_{m'k})R_{mm'}u_{m'l} + \sum_{m'}^{M}\sum_{l} D^{(2)}_{l}B^*_{m1}B_{m'2}\cosh(\theta_l)R_{mm'}\delta_{l=4\sim 8} \\
& + \sum_{m'}^{M}\sum_{i=1}^{3}\sum_{k=1}^{3} D^{(1)}_{ik}B^*_{mi}B_{m'i}\sinh(\theta_k)(\tilde{u}^*_{mk} + \tilde{u}_{m'k})R_{mm'}u_{m'l} + \sum_{m'}^{M}\sum_{l} D^{(3)}_{l}B^*_{m1}B_{m'3}\cosh(\theta_l)R_{mm'}\delta_{l=9\sim 13} \\
& + \sum_{m'}^{M}\sum_{k=4}^{8} D^{(2)}_{k}B^*_{m1}B_{m'2}(u^*_{mk}\cosh(\theta_k) + \tilde{u}_{m'l}\sinh(\theta_k))R_{mm'}u_{m'l} + \sum_{m'}^{M}\sum_{l} D^{(3)}_{l}B^*_{m3}B_{m'1}\cosh(\theta_l)R_{mm'}\delta_{l=19\sim 23} \\
& + \sum_{m'}^{M}\sum_{k=14}^{18} D^{(2)}_{k}B^*_{m1}B_{m'2}(u_{m'k}\cosh(\theta_k) + \tilde{u}^*_{mk}\sinh(\theta_k))R_{mm'}u_{m'l} \\
& + \sum_{m'}^{M}\sum_{k=4}^{8} D^{(2)}_{k}B^*_{m2}B_{m'1}(u^*_{m'k}\cosh(\theta_k) + \tilde{u}_{mk}\sinh(\theta_k))R_{mm'}u_{m'l} \\
& + \sum_{m'}^{M}\sum_{k=14}^{18} D^{(2)}_{k}B^*_{m2}B_{m'1}(u_{mk}\cosh(\theta_k) + \tilde{u}^*_{m'k}\sinh(\theta_k))R_{mm'}u_{m'l} \\
& + \sum_{m'}^{M}\sum_{k=9}^{13} D^{(3)}_{k}B^*_{m1}B_{m'3}(u^*_{mk}\cosh(\theta_k) + \tilde{u}_{m'k}\sinh(\theta_k))R_{mm'}u_{m'l} \\
& + \sum_{m'}^{M}\sum_{k=19}^{23} D^{(3)}_{k}B^*_{m1}B_{m'3}(u_{m'k}\cosh(\theta_k) + \tilde{u}^*_{mk}\sinh(\theta_k))R_{mm'}u_{m'l} \\
& + \sum_{m'}^{M}\sum_{k=9}^{13} D^{(3)}_{k}B^*_{m3}B_{m'1}(u_{m'k}\cosh(\theta_k) + \tilde{u}^*_{mk}\sinh(\theta_k))R_{mm'}u_{m'l} \\
& + \sum_{m'}^{M}\sum_{k=19}^{23} D^{(3)}_{k}B^*_{m3}B_{m'1}(u^*_{mk}\cosh(\theta_k) + \tilde{u}_{m'k}\sinh(\theta_k))R_{mm'}u_{m'l}
\end{aligned} \tag{S4}
$$

$$
\begin{aligned}
& i\sum_{i=0}^{3}\sum_{m'}^{M} B^*_{mi}B_{m'i}\dot{\tilde{u}}_{m'l}R_{mm'} + i\sum_{i=0}^{3}\sum_{m'}^{M}[B^*_{mi}\dot{B}_{m'i} + B^*_{mi}B_{m'i}(\sum_{k} u^*_{mk}\dot{u}_{m'k} + \sum_{k}\tilde{u}^*_{mk}\dot{\tilde{u}}_{m'k})]R_{mm'}\tilde{u}_{m'l} \\
&= \sum_{i=0}^{3}\sum_{m'}^{M} E_i B^*_{mi}B_{m'i}R_{mm'}\tilde{u}_{m'l} - \sum_{i=0}^{3} B^*_{mi}B_{m'i}\Omega_l\tilde{u}_{m'l}R_{mm'} + \sum_{m'}^{M}\sum_{i=1}^{3}\sum_{l=1}^{3} D^{(1)}_{il}B^*_{mi}B_{m'i}\sinh(\theta_l)R_{mm'}\delta_{l=1,2,3} \\
&+ \sum_{i=0}^{3}\sum_{m'}^{M} B^*_{mi}B_{m'i}\sum_{k}^{23}\Omega_k(u^*_{mk}u_{m'k} - \tilde{u}^*_{mk}\tilde{u}_{m'k})R_{mm'}\tilde{u}_{m'l} + \sum_{m'}^{M}(M_{\sigma_+}B^*_{m0}B_{m'1} + M^*_{\sigma_+}B^*_{m1}B_{m'0})R_{mm'}\tilde{u}_{m'l} \\
&+ \sum_{m'}^{M}\sum_{i=1}^{3}\sum_{k=1}^{3} D^{(1)}_{il}B^*_{mi}B_{m'i}\cosh(\theta_k)(u^*_{mk} + u_{m'k})R_{mm'}\tilde{u}_{m'l} + \sum_{m'}^{M}\sum_{l} D^{(2)}_{l}B^*_{m1}B_{m'2}\sinh(\theta_l)R_{mm'}\delta_{l=14\sim 18} \\
&+ \sum_{m'}^{M}\sum_{i=1}^{3}\sum_{k=1}^{3} D^{(1)}_{ik}B^*_{mi}B_{m'i}\sinh(\theta_k)(\tilde{u}^*_{mk} + \tilde{u}_{m'k})R_{mm'}\tilde{u}_{m'l} + \sum_{m'}^{M}\sum_{l} D^{(3)}_{l}B^*_{m1}B_{m'3}\sinh(\theta_l)R_{mm'}\delta_{l=19\sim 23} \\
&+ \sum_{m'}^{M}\sum_{k=4}^{8} D^{(2)}_{k}B^*_{m1}B_{m'2}(u^*_{mk}\cosh(\theta_k) + \tilde{u}_{m'l}\sinh(\theta_k))R_{mm'}\tilde{u}_{m'l} \\
&+ \sum_{m'}^{M}\sum_{k=14}^{18} D^{(2)}_{k}B^*_{m1}B_{m'2}(u_{m'k}\cosh(\theta_k) + \tilde{u}^*_{mk}\sinh(\theta_k))R_{mm'}\tilde{u}_{m'l} \\
&+ \sum_{m'}^{M}\sum_{k=9}^{13} D^{(3)}_{k}B^*_{m1}B_{m'3}(u^*_{mk}\cosh(\theta_k) + \tilde{u}_{m'l}\sinh(\theta_k))R_{mm'}\tilde{u}_{m'l} \\
&+ \sum_{m'}^{M}\sum_{k=19}^{23} D^{(3)}_{k}B^*_{m1}B_{m'3}(u_{m'k}\cosh(\theta_k) + \tilde{u}^*_{mk}\sinh(\theta_k))R_{mm'}\tilde{u}_{m'l} \\
&+ \sum_{m'}^{M}\sum_{l=1}^{3} D^{(1)}_{2l}B^*_{m2}B_{m'2}\sinh(\theta_l)R_{mm'}\delta_{l=1,2,3} + \sum_{m'}^{M}\sum_{k=1}^{3} D^{(1)}_{2k}B^*_{m2}B_{m'2}\cosh(\theta_k)(u^*_{mk} + u_{m'k})R_{mm'}\tilde{u}_{m'l} \\
&+ \sum_{m'}^{M}\sum_{k=1}^{3} D^{(1)}_{2k}B^*_{m2}B_{m'2}\sinh(\theta_k)(\tilde{u}^*_{mk} + \tilde{u}_{m'k})R_{mm'}\tilde{u}_{m'l} + \sum_{m'}^{M}\sum_{k=1}^{3} D^{(1)}_{3k}B^*_{m3}B_{m'3}\sinh(\theta_k)(\tilde{u}^*_{mk} + \tilde{u}_{m'k})R_{mm'}\tilde{u}_{m'} \\
&+ \sum_{m'}^{M}\sum_{k=4}^{8} D^{(2)}_{k}B^*_{m2}B_{m'1}(u^*_{mk}\cosh(\theta_k) + \tilde{u}_{m'l}\sinh(\theta_k))R_{mm'}\tilde{u}_{m'l} \\
&+ \sum_{m'}^{M}\sum_{k=14}^{18} D^{(2)}_{k}B^*_{m2}B_{m'1}(u_{m'k}\cosh(\theta_k) + \tilde{u}^*_{mk}\sinh(\theta_k))R_{mm'}\tilde{u}_{m'l} \\
&+ \sum_{m'}^{M}\sum_{k=1}^{3} D^{(1)}_{3k}B^*_{m3}B_{m'3}\cosh(\theta_k)(u^*_{mk} + u_{m'k})R_{mm'}\tilde{u}_{m'l} + \sum_{m'}^{M}\sum_{l} D^{(3)}_{l}B^*_{m3}B_{m'1}\sinh(\theta_l)R_{mm'}\delta_{l=19\sim 23} \\
&+ \sum_{m'}^{M}\sum_{k=9}^{13} D^{(3)}_{k}B^*_{m3}B_{m'1}(u^*_{mk}\cosh(\theta_k) + \tilde{u}_{m'l}\sinh(\theta_k))R_{mm'}\tilde{u}_{m'l} + \sum_{m'}^{M}\sum_{l=1}^{3} D^{(1)}_{3l}B^*_{m3}B_{m'3}\sinh(\theta_l)R_{mm'}\delta_{l=1,2,3} \\
&+ \sum_{m'}^{M}\sum_{k=19}^{23} D^{(3)}_{k}B^*_{m3}B_{m'1}(u_{m'k}\cosh(\theta_k) + \tilde{u}^*_{mk}\sinh(\theta_k))R_{mm'}\tilde{u}_{m'l} + \sum_{m'}^{M}\sum_{l} D^{(2)}_{l}B^*_{m2}B_{m'1}\sinh(\theta_l)R_{mm'}\delta_{l=14\sim 18}
\end{aligned}
\tag{S5}
$$
